# Mapping evidence on maternal metabolic conditions and child neurodevelopment in the Caribbean: a scoping review

**DOI:** 10.3389/fnut.2026.1686158

**Published:** 2026-03-16

**Authors:** April Mabie, Alexandra Van Cleave, Olivia Anne Foley, Caron Gray, Abubakar Tauseef, Jason Beste, Michelle María Jiménez de Tavárez

**Affiliations:** 1 Arrupe Global Scholars and Partnerships Program, Creighton University School of Medicine, Omaha, NE, United States; 2 Department of Obstetrics and Gynecology, Creighton University School of Medicine, Omaha, NE, United States; 3 Department of Internal Medicine, Creighton University, Omaha, NE, United States; 4 Arrupe Global Scholars and Partnerships Program, Creighton University School of Medicine, Phoenix, AZ, United States; 5 Facultad de Ciencias de la Salud, Pontificia Universidad Católica Madre y Maestra, Santiago De Los Caballeros, Dominican Republic

**Keywords:** autism spectrum disorder (ASD), Caribbean region, child neurodevelopment, maternal chemical exposure, maternal metabolic conditions (MMCs), maternal metabolism

## Abstract

**Background:**

Maternal metabolism and nutrition play a critical role in the healthy neurodevelopment of offspring during pregnancy. While numerous studies have established associations between maternal metabolic conditions (MMCs) and child neurodevelopment, the majority of this research has been conducted in high-income countries, particularly in the Global North. This scoping review aimed to identify and synthesize existing research on the relationship between MMCs and neurodevelopmental outcomes in children within the Caribbean region.

**Methods:**

This review was conducted following the methodological framework outlined by Arksey and O’Malley. A comprehensive search strategy was employed using MEDLINE (PubMed), EMBASE, and SCOPUS databases. In addition, gray literature was sourced through Google Scholar, hand-searching, and citation tracking.

**Results:**

A total of 970 articles were retrieved from the database searches, with an additional 34 identified as potential sources of gray literature and all 1,004 were screened. Following screening and eligibility assessment, 14 studies were included; 64.3% address maternal exposure to environmental chemicals, 50.0% describe the use of standardized cognitive assessments, and 21.4% of articles discuss the incidence of autism spectrum disorder (ASD) in offspring.

**Conclusion:**

There is a notable scarcity of research examining MMCs and child neurodevelopment within the Caribbean context. This gap necessitates the need for regional data generation and policy-informed research to better understand and address the unique maternal and child health challenges in the region.

**Systematic review registration:**

https://osf.io9ryja, identifier 9ryja.

## Introduction

Maternal metabolism and nutrition during pregnancy significantly impact the neurodevelopment of offspring (1). Maternal metabolic conditions (MMCs) that influence a child’s neurodevelopment include maternal weight, diabetes mellitus (DM), gestational diabetes mellitus (GDM), hypertension, metabolic syndrome, and exposure to xenobiotic chemicals (2–13). These influences include increased fetal exposure to inflammation as well as altered placental function (2). Sociodemographic and nutritional differences result in regional variations in metabolic profile. Therefore, the influences of MMCs on child neurodevelopment in the context of the Caribbean region is an important topic to explore.

Various cognitive tests exist to evaluate child neurodevelopment. These include “comprehensive neurodevelopment assessment tools [which] separately evaluate a child’s cognitive, motor, behavioral, and neurosensory capabilities,” and there are “standardized developmental assessments available for the purpose of testing development” (14). Previous studies have demonstrated the utility of various cognitive tests to evaluate the neurodevelopment of children from mothers experiencing MMCs (15–17).

Many studies identify the impact of maternal weight on the neurodevelopment of offspring, particularly in the development of attention deficit hyperactivity disorder (ADHD) and autism spectrum disorder (ASD) (2–7). Maternal obesity and consumption of a high fat diet are associated with an increased risk of developing both ADHD and ASD (2–7). The recent increase in ASD diagnoses throughout the past 40 years is connected to the increase in maternal metabolic syndrome prevalence, defined as a condition involving obesity, insulin resistance, and hyperlipidemia (12, 13). Additionally, a study conducted at the Cincinnati Children’s Hospital Medical Center found that both maternal obesity and GDM are associated with a 1.5-fold increased risk of having a child with ASD (8). A California study found that the MMCs of diabetes, hypertension, and obesity were all associated with an increased risk of ASD and other developmental delays (9).

Neurotoxic substances involve a wide array of materials, including air pollutants, heavy metals, and pesticides, among others (10). Maternal exposure to xenobiotic compounds impacts many organ systems in the mother, including endocrine and metabolic pathways (10). Studies investigating this exposure have demonstrated numerous associations between maternal exposure and subsequent neurodevelopment and behavioral disorders in children (10, 11).

Even though the connection between MMCs and child neurodevelopment has been well-established, most of the studies were conducted in the United States and Europe (18). It is important to evaluate the relevance and consistency of this topic in every area of the globe, especially among mothers and children from the Caribbean region, a historically under researched area (19). This scoping review aims to systematically map existing evidence on the association between MMC and child neurodevelopment in the Caribbean and to identify research gaps to guide future regional investigations.

## Methods

This scoping review of maternal metabolic impacts on child neurodevelopment in the Caribbean was conducted in accordance with the Arksey and O’Malley methodology for scoping reviews to increase transparency and rigor of the review process (20). The protocol for this review was registered in Open Science Framework (OSF) with registration link: https://doi.org/10.17605/OSF.IO/9RYJA. The PRISMA Extension for Scoping Reviews was used as the reporting guideline (21). Mendeley was used for citation management. Ethical approval was not required due to secondary data synthesis.

1. Identifying the Research Question

This scoping review aimed to answer the following research questions: What does the existing literature reveal about how a mother’s metabolic profile affects her child’s neurodevelopment in mothers and children from the Caribbean region? This research question was recommended by colleagues from the Dominican Republic who thought this to be a gap in the literature for this region.

2. Identifying Relevant Studies

**FIGURE 1:**
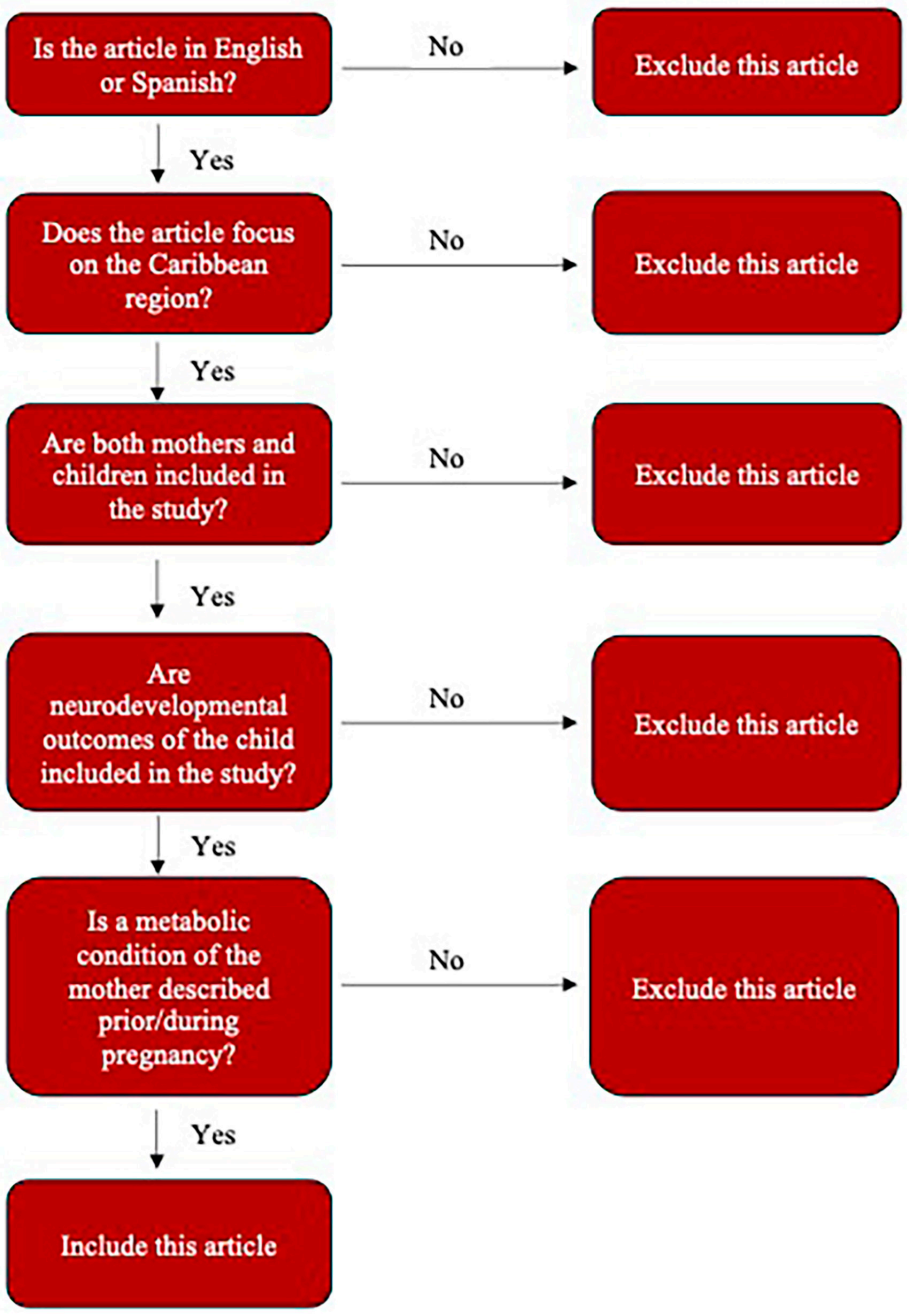
Inclusion and exclusion criteria flow chart. Figure was designed by authors.

Searches in MEDLINE (PubMed), EMBASE, and SCOPUS were conducted with the following the Participants-Concept-Context (PCC) inclusion criteria: participants include children, ages 0–19 years old of any gender, of mothers with an abnormal metabolic profile, concept is the effect of a mother’s abnormal metabolic profile, and the context is the Caribbean region. Inclusion and exclusion criteria, elaborated on in following paragraphs, can be seen in Figure 1.

Abnormal metabolic profile includes diagnoses such as obesity, insulin resistance, dyslipidemia, hypertension, and/or metabolic syndrome. This definition of abnormal metabolic profile is based on the concept of metabolic syndrome as defined by Swarup et al. (22). Articles describing xenobiotic compounds that produce these diagnoses in mothers were also included. This age range was chosen based on the Pan American Health Organization’s definition of pediatrics and adolescents to ensure broad inclusion (23, 24).

Neurodevelopment included information about developmental milestones, cognitive development, motor development, behavioral development, and language skills. These topics were chosen to broadly include a variety of different neurodevelopmental outcomes, as defined by Duncan and Matthews and the CDC (25, 26). Articles were excluded if they related to congenital defects, including neural tube defects.

Studies published in English and Spanish languages were included in this review. We included all studies, regardless of publication date as this is a scoping review and aims to capture all data on this subject. These language limitations were chosen based on the comprehension of our team. Sources of unpublished studies and gray literature searched included materials from the library at the Dominican medical school Pontificia Universidad Católica Madre y Maestra (PUCMM), Google Scholar, and by using citation tracing.

The context of this scoping review is the Caribbean region, as included by Medline “the area that lies between continental North and South America and comprises the Caribbean Sea, the West Indies, and the adjacent mainland regions of southern Mexico, Central America, Colombia, and Venezuela.” Countries included are Aruba, Caribbean Netherlands, Curacao, Saint Martin, Antigua and Barbuda, Bahamas, Barbados, the Virgin Islands, Cuba, Dominica, Dominican Republic, Grenada, Guadeloupe, Haiti, Jamaica, Martinique, Puerto Rico, Saint Kitts and Nevis, Saint Lucia, Saint Vincent and the Grenadines, and Trinidad and Tobago. Due to historical and cultural considerations, Suriname was included as well. Articles were excluded if they are solely written about the Global North/North America, Europe, Asia, Africa, Australia, or Antarctica.

AM, AV, and OF developed the three-step search strategy which aimed to locate both published and unpublished studies. First an initial limited search of MEDLINE (PubMed) and EMBASE was undertaken to identify articles on the topic. The text words contained in the titles and abstracts of relevant articles, and the index terms used to describe the articles were used to develop a full search strategy for MEDLINE (PubMed), EMBASE, and SCOPUS. Search strategy was developed with the help of Creighton University librarians Karina Kletscher and Judith Bergjord. It was not peer reviewed with PRESS; however, the PRESS checklist was referenced when creating the search strategy.

Briefly, the included search terms were maternal metabolic syndrome, maternal obesity, maternal diabetes mellitus, maternal hyperlipidemia, childhood neurodevelopment, childhood neurocognitive development, Caribbean region, West Indies, Dominican Republic, and Haiti. The search strategy, including all identified keywords and index terms, was adapted for each included database and/or information source. For full search strategy, see Supplementary Material 1. The reference list of all included sources of evidence were screened for additional studies. Final database searches were conducted October 8th, 2024 and all articles published prior to this date were included. Quality evaluation was not performed to prevent exclusion of available data.

3. Study Selection

**FIGURE 2:**
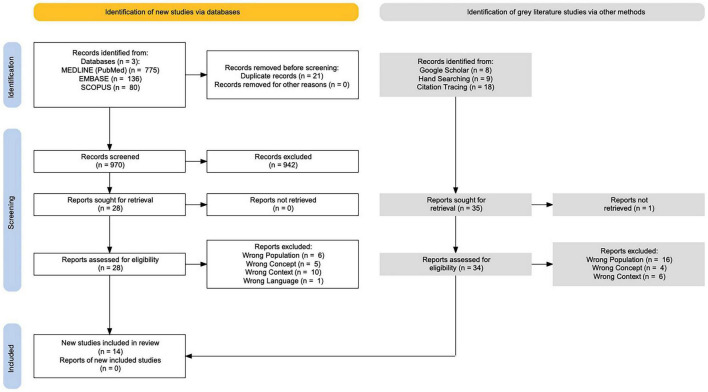
PRISMA diagram. Figure was developed using the PRISMA 2020 Guideline (21).

When selecting articles for this study, the eligibility criteria as stated above determined the article’s potential relevance. Articles were transferred directly into Rayyan from each database, and duplicates were removed. Two reviewers independently screened titles and abstracts against the eligibility criteria. Any disagreements were resolved via a third reviewer. The results of the search and study inclusion process are presented in a PRISMA diagram (see Figure 2).

Each article selected from the preliminary screening process was fully read by each of the three reviewers independently and data was gathered as described below. Studies excluded at that point were marked by which exclusion criteria they met. Any studies that were found via citation tracking and gray literature were evaluated by all three reviewers and accepted into this study via a majority consensus using the eligibility criteria.

4. Charting the Data

Data was extracted from the full articles by the three reviewers and regular discussions were held for any issues that arose. The following items were extracted: title, date of publication, source, study design, authors’ names, country of origin for the authors, principal outcome/research questions, location(s) investigated, type(s) of child neurodevelopment investigated, maternal metabolic conditions(s) investigated, main results, and study limitations.

5. Collating, summarizing and reporting the results

**FIGURE 3:**
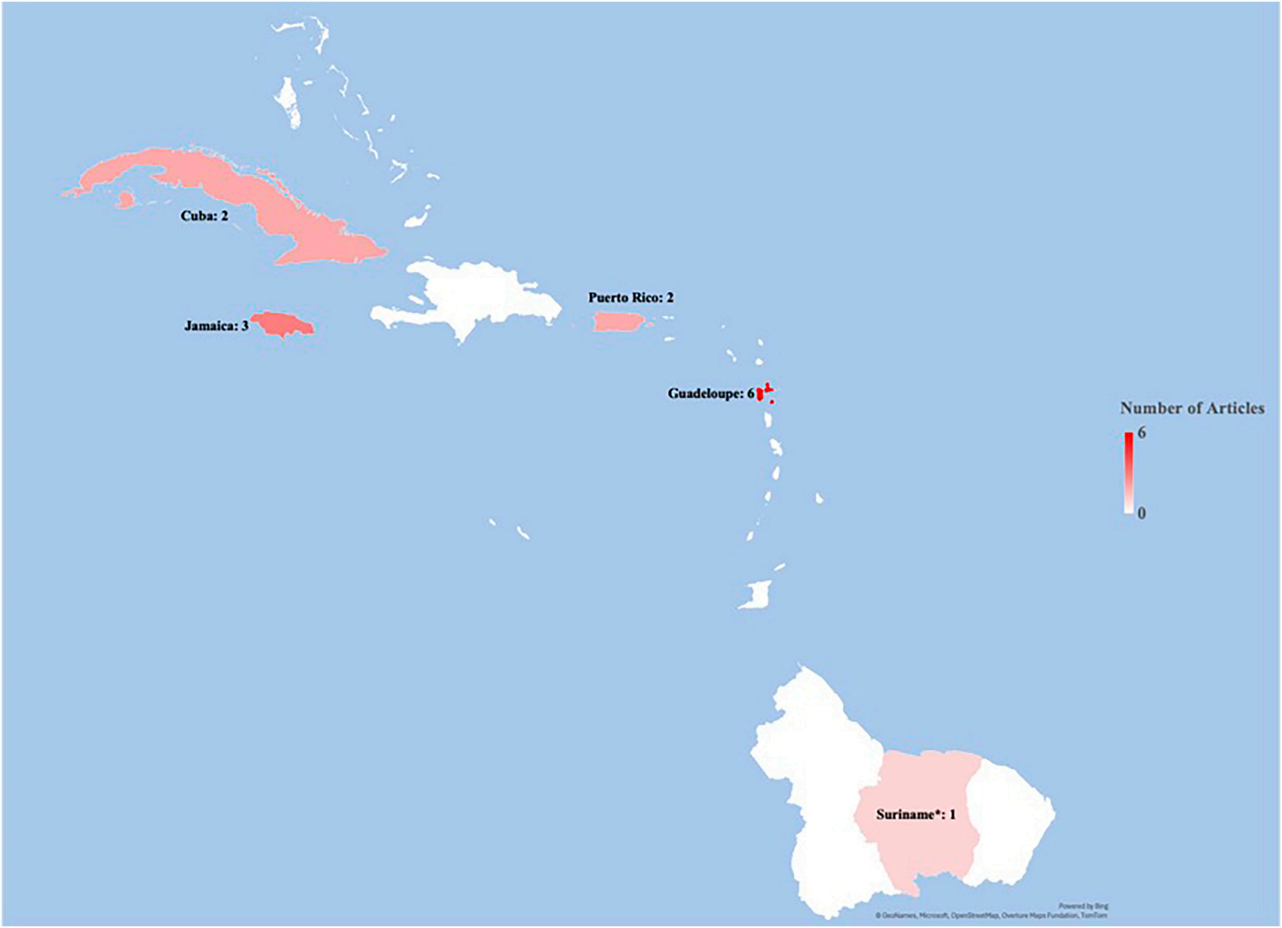
Location of included articles stratified by country. *Suriname, Guyana, and French Guiana are included as a part of the Caribbean region due to historical and cultural background.

A descriptive summary of the results and a qualitative thematic analysis was performed. A PRISMA flow diagram, seen in Figure 2, was used to demonstrate how articles were selected for inclusion/exclusion. The results are categorized by the following themes: Cognitive Tests, ASD, and Maternal Chemical Exposure, presented in Tables 1, 2. Another theme studied in this project was country investigated, and this data is presented in Figure 3.

**TABLE 1:** Descriptions of included studies.

References	Country	Maternal metabolic condition(s) investigated	Child neurodevelopment investigation	Study design (*n*)
Esquivel-Lauzurique et al. (27)	Cuba	Maternal nutrition during pregnancy	Early childhood educational programs about neurodevelopment, well child visits aimed at optimal growth and development	Perspective narrative
Hernández et al. (28)	Cuba	Diabetes mellitus, gestational diabetes, hypertension, hypothyroidism	Primary autism spectrum disorder diagnosis	Observational case-control study (*n* = 126 cases, *n* = 126 matched controls)
Boucher et al. (34)	Guadeloupe	Exposure to chlordecone: measured at birth from umbilical cord blood and breast milk sample 3 months postpartum	Adapted version of the Ages and Stages Questionnaire: 30 item questionnaire covers: personal-social, communication, problem-solving, fine motor, and gross motor	Prospective longitudinal cohort study (*n* = 141 umbilical cord samples) (*n* = 75 breast milk samples postpartum)
Cordier et al. (35)	Guadeloupe	Chlordecone measured in cord blood and breast milk samples	TSH, FT3 and FT4 determined in child blood at 3 months and toddlers assessed at 18 months using an adapted version of the Ages and Stages Questionnaire. All scores were then converted to IQ-like scores	Retrospective longitudinal cohort study (*n* = 111 children)
Saint-Amour et al. (36)	Guadeloupe	Chlordecone measured in cord blood and breast milk samples	Visual contrast sensitivity via the Freiburg Visual Acuity and Contrast Test (FrAcT)	Retrospective longitudinal cohort study (*n* = 285 chiIdren)
Desrochers-Couture et al. (37)	Guadeloupe	Chlordecone measured in cord blood and breast milk samples	Fine motor function via Bruininks Oseretsky Test of Motor Proficiency Second Edition (BOT-2). Postural hand tremor via Computerized Adaptive Testing System (CATSYS). Non-verbal visuospatial processing via Stanford Binet copying (S-B copying) test.	Ongoing, prospective cohort study (*n* = 410 children)
Oulhote et al. (38)	Guadeloupe	Concentrations of chlordecone and other environmental contaminants were measured in cord- and children’s blood	Cognitive abilities of children via the Wechsler Intelligence Scale for Children-IV (WISC-IV) [which are combined to obtain four composite scores in domains of verbal comprehension (Similarities And vocabulary), processing speed (Coding and Symbols), working memory (Letter-Number Sequencing and Digit Span), and perceptive reasoning (Block Design and Matrix Reasoning)]; Externalizing and internalizing problem behaviors via the Strengths and Difficulties Questionnaire (SDQ) completed by the child’s mother.	Prospective cohort study (*n* = 576 children)
Dallaire et al. (40)	Guadeloupe	Maternal cord chlordecone blood concentrations, breast milk chlordecone concentrations	Visual recognition memory and processing speed via the Fagan Tests of Infant Intelligence (FTID); visual acuity via the Teller Acuity Card; fine motor development via the Brunet-Lezine	Prospective cohort study (*n* = 153 infants) (*n* = 88 maternal cord blood and breast milk samples)
McCaw-Binns et al. (29)	Jamaica	Hypertension, weight gain, height, pre-eclampsia, diabetes	Developmental Milestones, Conners’ Teacher Rating Scale Score (ADHD/behavior), Child Behavior Checklist, Behavior and Emotional Rating Scale Score, SIPA Rating, WRAT Spelling, Reading and Arithmetic Score, Peabody Picture Vocabulary Test score, Ravens’ Progressive Matrices score.	Retrospective Cohort (Infants 6 wks–3 months *n* = 8567, 11–12 yo *n* = 1416, 15–16 yo *n* = 1343, 18–20 yo *n* = 790)
Walker et al. (30)	Jamaica	Pre-pregnancy BMI, Weight, height, and triceps skinfold test at every antenatal appointment	Raven’s Progressive Matrices, Peabody Picture Vocabulary Test, Digit Span Forward (a subtest of the Weschler Intelligence Scales)	Prospective Cohort (186)
Christian et al. (33)	Jamaica	Maternal Exposure to Pesticides	Development of Autism Spectrum Disorder	Retrospective Case-Control Study (*n* = 150 cases-control pain)
Jenkins et al. (39)	Puerto Rico	Maternal and prenatal exposure to glyphosate, measured through prenatal Aminomethylphosphonic acid (AMPA) concentrations (maternal urinary glyphosate analytes)	Adaptive, personal-social, communication, motor, and cognitive domains via the Battelle Developmental Inventory, 2nd edition Spanish (BD1-2) at 6/12/24 months	Prospective cohort study (*n* = 143 mother-child pairs)
Park et al. (31)	Puerto Rico	Phthalate metabolites were measured in multiple maternal urine collected during pregnancy.	Adaptive, personal-social, communication, motor, and cognitive domains via Battelle Developmental Inventory-2nd edition (BDI) at 6/12/24 months	Observational Cohort Study (*n* = 274 mother-child pairs)
Zijlmans et al. (32)	Suriname	Anthropometrics, blood pressure, pesticides (urine), maternal exposure history	Behavior and cognitive development, infant neuromotor development, autism spectrum disorder and neuropsychological development, Bayley Scales of Infant Development	Prospective Cohort (*n* = 1143 mothers, *n* = 992 children)

**TABLE 2:** Key findings from included studies.

References	Country	Title	Key findings
Esquivel-Lauzurique et al. (27)	Cuba	Comprehensive Care for Cuban Children in the First 1000 Days of Life	>90% of children aged 1 year in the Education Your Child program meeting indicators in all developmental spheres (intellectual, physical, socioaffective, and language)
Hernández et al. (28)	Cuba	Factores de riesgos heredofamiliares, prenatales, y perinatales en niños cubanos con autism primario [Family inherited, prenatal and perinatal factors in Cuban children with primary autism]	The odds of presenting autism were approximately 18 times in those born to mothers with a history of pregestational diabetes mellitus. DM prior to pregnancy was reported in 19 cases and only in 2 controls. Maternal hypertension during pregnancy presented in 24 cases and 11 controls. Both results were statistically significant. Gestational diabetes presented in 9 cases and 3 controls. Hypothyroidism was reported in 7 cases and 9 controls.
Boucher et al. (34)	Guadeloupe	Exposure to an organochlorine pesticide (chlordecone) and development of 18-months-old infants	ANOVAs reveal a significant effect of the chlordecone exposure group on the fine motor score. *Post hoc* least square difference analyses show that children with high chlordecone exposure obtained significantly lower scores than those with undetected cord chlordecone concentrations and those with low exposure levels. Results revealed that the relationship between higher chlordecone exposure and poorer fine motor scores is observed in boys only.
Cordier et al. (35)	Guadeloupe	Perinatal exposure to chlordecone, thyroid hormone status and neurodevelopment in infants: The Timoun cohort study in Guadeloupe (French West Indies)	Cord chlordecone associated with an increase in TSH in boys, whereas postnatal exposure was associated with a decrease in FT3 overall and in FT4 among girls. Higher TSH level at 3 months was positively associated with the ASQ score of fine motor development at 18 months among boys, but TSH did not modify the association between prenatal chlordecone exposure and poorer ASQ fine motor scores. Observed that among boys prenatal exposure to chlordecone was associated with increased level of TSH but did not seem to affect FT3 or FT4 levels whereas a decreased in FR3 was observed with postnatal exposure.
Saint-Amour et al. (36)	Guadeloupe	Visual contrast sensitivity in school-age Guadeloupean children exposed to chlordecone	Results showed that higher cord plasma chlordecone levels were associated with lower contrast sensitivity. A similar, although marginal, association was obtained after the additional statistical adjustment for the 7-years-old child exposure. Although child chlordecone levels was not associated with the FrAcT, sex-specific stratified analyses revealed significant associations in boys. This study indicates that exposure to chlordecone *in utero* and during childhood may impair visual contrast sensitivity at school age, particularly in boys. A decrease of contrast sensitivity can be due to alterations of ocular and/or retinal/brain processing.
Desrochers-Couture et al. (37)	Guadeloupe	Visuospatial processing and fine motor function among 7-years old Guadeloupe children pre-and postnatally exposed to the organochlorine pesticide chlordecone	Cord chlordecone concentrations are associated with regular frequency pattern of subtle hand tremors in both hands, and not related visual processing and fine motor precision. Chlordecone concentrations in blood sample were associated with poorer visual processing when copying geometric figures, but not related to poorer fine movement precision in tasks requiring pencil, scissors, and paper. No sex-specific differences.
Oulhote et al. (38)	Guadeloupe	Prenatal and childhood chlordecone exposure, cognitive abilities and problem behaviors in 7-years-old children: the TIMOUN mother-child cohort in Guadeloupe	A twofold increase in cord blood concentrations was associated with 0.05 standard deviation (SD) higher internalizing problem scores, whereas 7-years chlordecone concentrations were associated with lower Full-Scale IQ scores (FSIQ) and greater externalized behavioral problem scores. A twofold increase in 7-years chlordecone concentrations was associated with a decrease of 0.67 point on FSIQ and an increase of 0.04 SD on externalizing problems. These associations with Cognitive abilities were driven by decreases in perceptive reasoning, working memory and verbal comprehension. Associations between 7-years exposure and perceptive reasoning, working memory, and the FSIQ were stronger in boys, whereas cord blood and child blood associations with internalizing problems were stronger in girls.
Dallaire et al. (40)	Guadeloupe	Cognitive, visual, and motor development of 7-months-old Guadeloupean infants exposed to chlordecone	Cord chlordecone concentrations in tertiles were associated with reduced novelty preference on the FTII in the highly exposed group. Postnatal exposure through contaminated food consumption was marginally related to reduced novelty preference and longer processing speed. Detectable levels of chlordecone in cord blood were associated with higher risk of obtaining low scores on the fine motor development scale.
McCaw-Binns et al. (29)	Jamaica	Cohort Profile: The Jamaican 1986 Birth Cohort Study	Perinatal study documented incidence and causes of perinatal deaths and the influence of social and environmental risk factors, maternal behavior, poor obstetric history, medical complications and poor quality of antenatal and intrapartum care.
Walker et al. (30)	Jamaica	Association of growth *in utero* with cognitive function at age 6–8 years	Maternal weight and BMI in early pregnancy were not associated with the children’s cognitive scores. There were also no significant differences when the mothers were categorized by BMI as underweight, normal weight, overweight or obese. Weight gain was also not significantly associated with the cognitive scores. Change in triceps skinfold from 25 to 35 weeks gestation was positively correlated with the children’s scores on the Raven’s test
Christian et al. (33)	Jamaica	Maternal exposures associated with autism spectrum disorder in Jamaican children	Maternal exposure to pesticides was also associated with ASD in the children in the crude model and after adjusting for the child’s parish
Jenkins et al. (39)	Puerto Rico	Gestational glyphosate exposure and early childhood neurodevelopment in a Puerto Rico birth cohort	Prenatal AMPA concentrations were negatively associated with communication domain at 12 months and communication subdomain scores at 12 and 24 months. At 24 months, four BDI-2 domains were associated with AMPA: adaptive, personal-social, communication, and cognitive. Similar trends were observed with GLY concentrations, but most confidence intervals include zero. We found no significant associations at 6 months. Our results suggest that gestational exposure to glyphosate is associated with adverse early neurodevelopment, with more pronounced delays at 24 months
Park et al. (31)	Puerto Rico	Gestational exposure to phthalates and phthalate replacements in relation to neurodevelopmental delays in early childhood	Results show that aIl five domains were significantly associated with mono-3-carboxypropyl phthalate (MCPP) at age 24 months, suggesting a holistic developmental delay related to this metabolite.
Zijlmans et al. (32)	Suriname	Caribbean Consortium for Research in Environmental and Occupational Health (CCREOH) Cohort Study: Influences of Complex Environment Exposures on Maternal and Child Health in Suriname	Pesticide residues exceeding European union maximum residue limits, including prohibited worldwide endosulfan and lindane in the leafy vegetable tannia. Tannia was the most frequently consumed (89.3%); 36.5% participants had high intake rates of tannia (>36 g/day). Tannia is also a commonly used vegetable in baby food preparation in Suriname. Ongoing study regarding neurodevelopment outcomes of children

## Results

A total of 14 articles were included in scoping review analysis. Six were found from database searching while eight were identified from gray literature searching (27–40). Gray literature was located via Google Scholar and citation tracing and included strictly peer-reviewed studies. The 14 included studies were conducted in the following countries: 2 in Cuba, 3 in Jamaica, 2 in Puerto Rico, 6 in Guadeloupe, and 1 in Suriname. The geographical breakdown of the studies included is demonstrated in Figure 3. The ages of the children included in the studies range from birth to 19 years. Of the included studies, there were 7 prospective cohort studies, 3 retrospective cohort studies, 1 observational cohort study, 1 observational case-control, 1 retrospective case-control, and 1 perspective narrative. Three key themes that emerged among included articles were congruences of neurodevelopmental tests, ASD development, and maternal chemical exposure. Other key findings of the studies included can be found in Tables 1, 2. Two systematic reviews and one scoping review were found, but they included data mostly from Latin American and not the Caribbean (17, 41, 42). Thus, they were utilized to locate seven additional articles that were included in the study via citation tracing (34–40).

One of the 14 included articles by Zijlmans et al. detailing research conducted in Suriname. Although Suriname is technically located in South America, it was included in this study because historically, geographically, and culturally, the Guianas (Suriname, French Guiana, and Guyana), are considered by some as part of the Caribbean due to geographical isolation and history of colonialism (43). Upon review of physical copies of research theses conducted by medical students in the library at PUCMM’s Santiago campus, several studies were identified investigating the metabolic health of mothers. However, there were no studies directly linking maternal metabolic health with child neurodevelopment, so these non-peer reviewed studies were excluded.

### Congruences of neurodevelopmental tests

Seven of fourteen studies discussed neurodevelopmental testing. Two studies of Guadeloupe investigated the outcomes of childhood neurodevelopment using the Ages and Stages Questionnaire at 18 months (34, 35). Two different studies investigated the cognitive development of children using the Wechsler Intelligence Scale, one from Guadeloupe and one from Jamaica (30, 38). Two of the Jamaican studies included both the Ravens’ Progressive Matrices Score and the Peabody Picture Vocabulary Test score (29, 30). Both studies from Puerto Rico used the Battelle Developmental Inventory (31, 39).

### ASD development

Of the fourteen included articles, three studied the relationship between development of ASD and a maternal metabolic condition (28, 32, 33). Two of these studies, one conducted in Jamaica and the other Suriname, examined the connection between ASD development and maternal exposure to pesticides (32, 33). One study conducted in Cuba examined the relationship between ASD development and maternal DM, GDM, hypertension and hypothyroidism (28).

### Maternal chemical exposure

Nine of fourteen studies investigated neurodevelopmental outcomes related xenobiotic exposure, metabolically active chemicals. Six of these were from one cohort in Guadeloupe which examined chlordecone exposure (34–38, 40). Three studies evaluated specific xenobiotic chemicals, including products containing volatile organic compounds and pesticides, glyphosate and aminomethylphosphonic acid, and phthalates (31, 33, 39).

## Discussion

### Summary of main findings

This is the first scoping review that analyzes the impact maternal metabolism and nutrition have on neurodevelopment of offspring in the Caribbean region. Overall, three key themes emerged from this scoping review, including cognitive tests used, risk factors for ASD, and the impact of maternal chemical exposure.

### Comparison with global evidence

Studies from the Global North examining child neurodevelopment utilize a variety of cognitive tests. These cognitive tests include the McCarthy Scales of Children’s Abilities, the Hammersmith Infant Neurological Exam, and the British Picture Vocabulary Scale (15, 16, 44). The Bayley Scales of Infant and Child Development has been used in studies from both the Global North and the Caribbean, demonstrating global recognition of this cognitive test (16, 32). Uniquely, aspects of the Wechsler Intelligence Scale were utilized in two included articles in this study that were conducted in two separate Caribbean countries, suggesting regional preference of this cognitive test (30, 38). A scoping review published in Spain in 2015 also identified the Wechsler Intelligence Scale as a common cognitive assessment for offspring of women with MMCs (17). This demonstrates some similarities in cognitive test use across the globe, despite a lack of overall standardization.

Another theme that emerged throughout this review was development of ASD in offspring. The prevalence of ASD is increasing worldwide, which may have driven the desire to measure this specific outcome (45). One study conducted in Cuba found that an ASD diagnosis was 18 times more likely if the mother suffered from DM when compared to those without DM and was also significantly more likely if mothers suffered from hypertension during pregnancy (28). A study from Jamaica also found that maternal exposure to pesticides positively correlated with an ASD diagnosis (33). This topic has been studied extensively throughout the Global North and recent studies have demonstrated a clear connection between MMCs and a diagnosis of ASD (9, 46–58). Although this demonstrates that findings from studies conducted in the Caribbean region are similar to those from the Global North, the difference in study quantity sets the two areas apart.

A predominant theme observed in nine of the fourteen articles was investigation of maternal chemical exposure and its impact on a child’s neurodevelopment. Six of the nine articles came from the mother-child TIMOUN cohort in Guadeloupe, a cohort of neonates exposed *in utero* and during childhood to the chemical chlordecone (34–38, 40). At the outset of this study, the intention was not to explore chemical exposures as the concept of interest was maternal metabolic profile. However, in a paper by Hervé et al. describing the intent of the TIMOUN cohort studies, chlordecone is described as an organochlorine insecticide which functions as an endocrine-disrupting chemical which interacts with both estrogen and progesterone receptors (59). Due to the metabolic influence of this chemical, further review of these articles was warranted.

Chlordecone, a chemical insecticide used on tobacco, bananas, and citrus trees, has not been manufactured or used since the late 1970’s in the US (60). The Food and Drug Administration (FDA) has not found it in food during studies across the US since 1992 (60). Between 1973 and 1993, chlordecone was used extensively in the French West Indies, specifically Guadeloupe and Martinique, as an insecticide for banana farms (61). As this chemical undergoes significantly slow degradation, it remains present in the soil long after application (61). Few human studies on chlordecone have been written in the Global North. One study from Hopewell, Virginia explored the chemical’s effects on people exposed after chemical dumping along the James River (62, 63). However, this exposure primarily impacted men, and no studies were done on pregnant women or their children (61, 62).

### Implications for regional research

The geographic distribution of these studies may reflect the priorities of the research being conducted in the Caribbean region. These results may indicate that the consequences of chemical exposure might be of greater interest to this region as compared to investigating chronic conditions, such as metabolic syndrome. While chemical exposures are important, the impact of chronic MMCs should be further explored in this region to provide justification for preventative health interventions for pregnant people with these metabolic conditions.

Although data on MMCs and child neurodevelopment in the Caribbean was found, it was highly concentrated in only a few countries throughout the region. The TIMOUN cohort and the PROTECT cohort provide data for all included studies from Guadeloupe and Puerto Rico, respectively. This suggests an interest in the connection between MMCs and child neurodevelopment but produces results with limited regional generalizability. Moreover, of the 21 countries included in the initial eligibility criteria, relevant studies were found in only four countries. It is important to conduct further research on this topic throughout the Caribbean to create a more accurate picture of how MMCs are impacting child neurodevelopment in this region. Further emphasis must also be placed on identifying culturally relevant data rather than relying on the work of researchers from the Global North. Of the 14 articles included in this study, 9 were conducted with at least one author, but in some cases all authors, from the Global North (31, 33–40).

As current regional evidence is predominantly published by authors based within the Global North, future work should feature greater inclusion of Global South colleagues who may currently be limited by socioeconomic constraints, including lack of funding and language barriers. Partnerships would allow for growth of the existing evidence base with stronger cultural and regional understanding. By collaborating globally, teams can prioritize the development of internationally standardized neurodevelopment tools and identification of metabolomic biomarkers with potential for future screening options.

### Strengths and limitations

This scoping review further demonstrates that despite paucity of published research on MMCs and child neurodevelopment in the Caribbean, a greater amount of unpublished work may exist. The authors found an extensive library of unpublished research at PUCMM, a regional medical school in the Dominican Republic. A regional school is defined as a medical school that trains students to practice in the region where the school is located, rather than training students for practice in the United States or Canada (64). There are currently 43 regional medical schools in the Caribbean region: 1 in Barbados, 14 in Cuba, 10 in the Dominican Republic, 1 in Guadeloupe, 1 in Guyana, 9 in Haiti, 1 in Jamaica, 4 in Puerto Rico, 1 in Suriname, and 1 in Trinidad and Tobago (64).

Although none of the studies found in the PUCMM fit the inclusion criteria for this study, it is possible that relevant, unpublished research exists in other regional medical schools throughout the Caribbean. This highlights a major accessibility issue hampering the study of MMCs and child neurodevelopment in this region. It is thus necessary to investigate what research regarding this topic already exists in the Caribbean and to promote further study of MMCs and child neurodevelopment if none is found.

## Limitations

There are several limitations of this study. As a scoping review, there is an essential limitation that scoping reviews do not “appraise quality of evidence” (20). Therefore, this study does not investigate the quality of the articles included, nor does it provide any comment on it. Moreover, this is not an exhaustive search of all the gray literature, digitized and non-digitized, that may exist in each country. Though an extensive searching strategy was developed, it is impossible to generate all relevant search terms that exist, so some articles may have been missed. A significant portion of countries in the Caribbean are not Spanish or English speaking, and as such, some relevant articles in excluded languages may have been missed, introducing publication bias. Therefore, future research should explore this topic in languages outside of Spanish and English, including French, Dutch, and other official languages of the Caribbean. As mentioned, the library at PUCMM demonstrated the large quantity of unpublished literature that exists in physical copies only at the school’s campus. Therefore, a limitation of this study was the lack of accessibility to research in other regional medical schools throughout the Caribbean region. Notably, only fourteen articles were included in this study, with variable sample sizes and non-uniform measures, limiting generalizability.

## Conclusion

This scoping review highlights a significant paucity of Caribbean research on maternal metabolic conditions and child neurodevelopment. Of existing literature, the primary themes identified were neurodevelopment tests, ADS development, and maternal chemical exposure. The majority of included articles focused on maternal chemical exposure, and all articles originated from only four countries in the Caribbean region. Moving forward, strengthening regional data generation and improving access to unpublished work will be critical to developing targeted interventions and policies.

## Data Availability

The original contributions presented in this study are included in this article and original search strategy is provided in the Supplementary material. Further inquiries can be directed to the corresponding authors.

## Supplementary material

Supplementary Material 1 Full search strategy used in MEDLINE (PubMed), EMBASE, and SCOPUS.
