# Supplementary material for: Mapping evidence on maternal metabolic conditions and child neurodevelopment in the Caribbean: a scoping review
Source: Front Nutr. 2026 Mar 16;13:1686158. doi: 10.3389/fnut.2026.1686158 (PMC13033538; doi:10.3389/fnut.2026.1686158)
Supplement: Supplementary Material 1 — Full search strategy used in MEDLINE (PubMed), EMBASE, and SCOPUS. [file Table_1.docx]

**Below, please find the detailed search terms used to conduct this scoping review. By using the PCC model, the three areas were combined using “AND.”**

**Embase:**

Population Search Terms:

('maternal welfare'/exp OR 'maternal child health' OR 'maternal child welfare' OR 'maternal health' OR 'maternal welfare' OR 'welfare, maternal' OR 'prenatal period' OR 'perinatal period' OR 'maternal medicine' OR 'dyad mother–child' OR (dyad AND mother–child) OR 'mother child relation'/exp OR 'maternal child health care' OR 'pregnancy outcome'/exp OR 'pregnancy outcome') AND ('hypertension'/exp OR 'htn (hypertension)' OR 'acute hypertension' OR 'arterial hypertension' OR 'blood pressure, high' OR 'cardiovascular hypertension' OR 'controlled hypertension' OR 'endocrine hypertension' OR 'high blood pressure' OR 'high renin hypertension' OR 'hypertension' OR 'hypertensive disease' OR 'hypertensive effect' OR 'hypertensive reaction' OR 'hypertensive response' OR 'neurogenic hypertension' OR 'preexistent hypertension' OR 'salt high blood pressure' OR 'salt hypertension' OR 'secondary hypertension' OR 'systemic hypertension' OR 'hyperglycemia'/exp OR 'glucose blood level, elevated' OR 'glycemia, hyper' OR 'high blood glucose' OR 'hyper-glycaemia' OR 'hyper-glycemia' OR 'hyperglucemia' OR 'hyperglycaemia' OR 'hyperglycaemias' OR 'hyperglycemia' OR 'hyperglycemias' OR 'hyperglycemic syndrome' OR 'diabetes mellitus'/exp OR 'diabetes' OR 'diabetes mellitus' OR 'diabetic' OR 'diabets' OR 'unspecified diabetes mellitus' OR 'lipid disorder'/exp OR 'metabolism'/exp OR 'cometabolism' OR 'conversion, metabolic' OR 'intermediary metabolism' OR 'metabolic networks and pathways' OR 'metabolic pathway' OR 'metabolic phenomena' OR 'metabolic phenomenon' OR 'metabolic response' OR 'metabolic route' OR 'metabolic state' OR 'metabolism' OR 'metabolization' OR 'nutrition'/exp OR 'diet, food, and nutrition' OR 'nutrition' OR 'nutrition council' OR 'nutrition phenomena' OR 'nutrition physiology' OR 'nutrition processes' OR 'nutrition research' OR 'nutrition research center' OR 'nutrition study' OR 'nutrition survey' OR 'nutrition surveys' OR 'nutritional physiological phenomena' OR 'nutritional physiology' OR 'nutritive solution' OR 'sports nutritional physiological phenomena' OR 'overnutrition' OR 'metabolic regulation' OR 'nutritional and metabolic disorder'/exp OR 'metabolic and nutritional disease' OR 'metabolic and nutritional diseases' OR 'metabolic and nutritional disorder' OR 'metabolic and nutritional disorders' OR 'nutritional and metabolic disease' OR 'nutritional and metabolic diseases' OR 'nutritional and metabolic disorder' OR 'nutritional and metabolic disorders' OR 'metabolic syndrome x'/exp OR 'metsyn (metabolic syndrome)' OR 'reaven syndrome' OR 'reaven syndrome x' OR 'reaven`s syndrome' OR 'reaven`s syndrome x' OR 'insulin resistance syndrome' OR 'metabolic x syndrome' OR 'metabolic syndrome' OR 'metabolic syndrome x' OR 'syndrome x, metabolic' OR 'syndrome of insulin resistance' OR 'anthropometry'/exp OR 'anthropometric index' OR 'anthropometric measurement' OR 'anthropometrics' OR 'anthropometry' OR 'antropometry' OR 'body measurement') OR 'maternal obesity'/exp OR 'maternal obesity' OR 'mother`s obesity' OR 'obesity in pregnancy' OR 'obesity of pregnancy' OR 'obesity, maternal' OR 'pregnancy-associated obesity' OR 'pregnancy-related obesity' OR 'gestational diabetes'/exp OR 'diabetes mellitus gravidarum' OR 'diabetes mellitus of pregnancy' OR 'diabetes of pregnancy' OR 'diabetes, gestational' OR 'diabetes, pregnancy' OR 'gestational diabetes' OR 'gestational diabetes mellitus' OR 'maternal gestational diabetes mellitus' OR 'pregnancy diabetes' OR 'pregnancy diabetes mellitus' OR 'pregnancy-induced diabetes' OR 'maternal hypertension'/exp OR 'pih (pregnancy induced hypertension)' OR 'gestational hypertension' OR 'hypertension during pregnancy' OR 'hypertension in pregnancy' OR 'hypertension induced by pregnancy' OR 'hypertension, maternal' OR 'hypertension, pregnancy induced' OR 'hypertension, pregnancy-induced' OR 'hypertensive disorder of pregnancy' OR 'maternal hypertension' OR 'pregnancy associated hypertension' OR 'pregnancy hypertension' OR 'pregnancy induced hypertension' OR 'pregnancy-induced hypertension' OR 'maternal hyperglycemia'/exp OR 'maternal metabolism'/exp OR 'maternal nutrition'/exp OR 'diet, maternal' OR 'maternal diet' OR 'maternal nutrition' OR 'maternal nutrition physiology' OR 'maternal nutritional physiological phenomena' OR 'nutrition, maternal' OR 'prenatal nutrition' OR 'prenatal nutrition physiology' OR 'prenatal nutritional physiological phenomena' OR 'prenatal nutritional physiological phenomenon'

Concept:

'autism'/exp OR 'kanner syndrome' OR 'pdd (pervasive developmental disorder)' OR 'autism' OR 'autism spectrum disorder' OR 'autism, early infantile' OR 'autism, infantile' OR 'autistic child' OR 'autistic children' OR 'autistic disorder' OR 'autistic spectrum disorder' OR 'child autism' OR 'child autism rating scale' OR 'child development disorders, pervasive' OR 'childhood autism' OR 'classical autism' OR 'early infantile autism' OR 'infantile autism' OR 'infantile autism, early' OR 'pervasive child development disorders' OR 'pervasive developmental disorder' OR 'pervasive developmental disorders' OR 'typical autism' OR 'attention deficit hyperactivity disorder'/exp OR 'adhd' OR 'attention deficit' OR 'attention deficit and disruptive behavior disorders' OR 'attention deficit and disruptive behavior disorders' OR 'attention deficit disorder' OR 'attention deficit disorder with hyperactivity' OR 'attention deficit hyperactivity disorder' OR 'intelligence quotient'/exp OR 'i.q' OR 'i.q.' OR 'intellectual quotient' OR 'intelligence coefficient' OR 'intelligence quotient' OR 'intelligent quotient' OR 'iq' OR 'iq score' OR 'quotient, intelligence' OR 'working memory index'/exp OR 'processing speed index'/exp OR 'perceptual reasoning index'/exp OR 'verbal comprehension index'/exp OR 'psychological rating scale'/exp OR 'psychological rating scale' OR 'wechsler intelligence scale for children'/exp OR 'wechsler intelligence scale for children' OR 'bayley scales of infant development' OR 'children neurodevelopment' OR 'developmental neurology & neurodisability' OR 'developmental neurology' OR neurodisability OR ‘infant neurodevelopment’ OR (('children'/exp OR children) AND ('neurodevelopment'/exp OR neurodevelopment)) OR 'youth neurodevelopment' OR (('youth'/exp OR youth) AND ('neurodevelopment'/exp OR neurodevelopment)) OR 'child neurodevelopment' OR (('child'/exp OR child) AND ('neurodevelopment'/exp OR neurodevelopment)) OR 'nerve cell differentiation' OR 'developmental milestones' OR (developmental AND milestones) OR 'disorders of higher cerebral function'/exp OR 'disorders of higher cerebral function' OR 'early child development' OR (early AND ('child'/exp OR child) AND ('development'/exp OR development)) OR 'cognition' OR learning OR 'cognitive development'/exp OR 'brain development' OR ‘mental performance’ OR ‘motor performance’ OR 'cognitive development' OR 'motor development'/exp OR 'motor development' OR 'psychosocial development'/exp OR 'psychosocial development' OR 'emotional development'/exp OR 'emotional development' OR 'language development'/exp OR 'language development' OR 'language disability' OR 'developmental language disorder'/exp OR 'developmental language disorder' OR 'psychomotor development'/exp OR 'development, psychomotor' OR 'development, psychomotoric' OR 'psychomotor ability' OR 'psychomotor development' OR 'psychomotor evolution' OR 'psychomotoric development' OR 'psychomotor development index'/exp OR 'executive function test' OR ‘executive function’

Context:

'aruba'/exp OR 'aruba' OR 'caribbean netherlands'/exp OR 'caribbean netherlands' OR 'curacao'/exp OR 'curacao' OR 'saint martin (dutch)'/exp OR 'saint martin (dutch)' OR 'antigua and barbuda'/exp OR 'antigua and barbuda' OR 'bahamas'/exp OR 'bahamas' OR 'barbados'/exp OR 'barbados' OR 'virgin islands (british)'/exp OR 'virgin islands (british)' OR 'cuba'/exp OR 'cuba' OR 'cuban'/exp OR 'cuban' OR 'dominica'/exp OR 'dominica' OR 'dominican (dominica)'/exp OR 'dominican (dominica)' OR 'dominican republic'/exp OR 'dominican republic' OR 'dominican (dominican republic)'/exp OR 'dominican (dominican republic)' OR 'grenada'/exp OR 'grenada' OR 'guadeloupe'/exp OR 'guadeloupe' OR 'haiti'/exp OR 'haiti' OR 'haitian'/exp OR 'haitian' OR 'jamaica'/exp OR 'jamaica' OR 'jamaican'/exp OR 'jamaican' OR 'martinique'/exp OR 'martinique' OR 'puerto rico'/exp OR 'puerto rico' OR 'puerto rican'/exp OR 'puerto rican' OR 'saint kitts and nevis'/exp OR 'saint kitts and nevis' OR 'saint lucia'/exp OR 'saint lucia' OR 'saint vincent and the grenadines'/exp OR 'saint vincent and the grenadines' OR 'trinidad and tobago'/exp OR 'trinidad and tobago' OR 'virgin islands (u.s.)'/exp OR 'virgin islands (u.s.)' OR 'caribbean islands'/exp OR 'caribbean islands' OR 'caribbean'/exp OR 'caribbean' OR 'caribbean (person)'/exp OR 'caribbean (person)'

**MEDLINE:**

Population:

(MH "Obesity, Maternal") OR maternal n1 obesity OR (MH "Diabetes, Gestational") OR gestation* n1 diabetes OR (MH "Pregnancy in Diabetics+") OR pregnancy n1 diabetic OR pregnancy n1 diabetes OR (MH "Hypertension, Pregnancy-Induced") OR pregnancy n1 hypertension OR pregnancy n1 hypertensive OR (MH "Prenatal Nutritional Physiological Phenomena") OR prenatal n1 nutrition OR ((MH "Lipid Metabolism Disorders+") OR lipid n1 metabolism OR lipid n1 disorder* OR (MH "Hyperglycemia+") OR (MH "Diabetes Mellitus, Type 2+") OR (MH "Insulin Resistance+") OR (MH "Metabolic Syndrome") OR metabolism OR metabolic AND (MH "Maternal Health") )

Concept:

( ((MH "Language Development+") OR language n1 development OR language OR development) AND ( (MH "Pediatrics+") OR ( (MH "Adolescent Development") OR adolescent n1 development OR (MH "Child Development+") OR child n1 development ) OR ( (MH "Growth and Development+") OR growth OR development ) OR ( (MH "Child") OR (MH "Child Health") OR child n1 health ) ) ) OR ( ( (MH "Psychosocial Functioning") OR psychosocial OR psychosocial n1 function* OR (MH "Functional Status") OR functional OR ( (MH "Neurodevelopmental Disorders+") OR neurodevelopmental n1 disorders OR (MH "Neurocognitive Disorders+") OR neurocognitive) OR ( (MH "Psychomotor Performance+") OR psychomotor OR (MH "Mental Processes+") OR mental n1 processes OR (MH "Mental Competency") OR mental OR competency OR competencies )

Context:

(MH "Caribbean Region+") OR West n1 Indies OR Dominican n1 Republic OR Haiti OR Dominican OR Haitian

**Scopus:**

Population:

((prenatal OR peripartum OR maternal OR "maternal health" OR "maternal welfare" OR gestation* OR antenatal OR preconcept* OR "maternal child health" OR "maternal child welfare" OR "welfare, maternal" OR "peripartum" ) AND ( obesity OR "heart disease" OR "metabolic syndrome" OR atherosclerosis OR hypertensi* OR "acute hypertension" OR "blood pressure, high" OR "diabetes mellitus" OR diabetes OR hyperglyc* OR "insulin resistance" OR nutrition OR "glucose intolerance" OR hypercholesterolemia OR dyslipidemia OR hyperlipidemia OR "lipid metabolism disorders" OR metaboli* OR htn OR "arterial hypertension" OR hyperinsulinism )) OR ("maternal obesity" OR "obesity in pregnancy" OR "obesity, maternal" OR "pregnancy-associated obesity" OR "pregnancy-related obesity" OR "gestational diabetes" OR "maternal hypertension" OR "pregnancy-induced hypertension" OR "gestational hypertension" OR "hypertension in pregnancy" OR "hypertension induced by pregnancy" OR "hypertension, maternal" OR "hypertension, pregnancy" OR "hypertensive disorder of pregnancy" OR "maternal hyperglyc*" OR "maternal metabolism" OR "maternal nutrition" OR "diet, maternal" OR "maternal diet" OR "prenatal nutrition" OR "prenatal metabolism" OR "antenatal nutrition" OR "antenatal metabolism" OR "maternal heart disease" OR "maternal atherosclerosis" OR "pregnancy metabolism" OR "maternal diabetes mellitus" OR "maternal hypercholesterolemia" OR "maternal hyperlipidemia" OR "maternal dyslipidemia" OR "maternal insulin resistance" OR "maternal hyperinsulinism" OR "pih (pregnancy induced hypertension)" OR "hypertension during pregnancy" OR "hypertension, pregnancy induced" OR "hypertension, pregnancy-induced" OR "maternal hypertension" OR "pregnancy associated hypertension" OR "pregnancy hypertension" OR "pregnancy induced hypertension" OR "maternal nutrition physiology" OR "maternal nutritional physiological phenomena" OR "nutrition, maternal" OR "prenatal nutrition physiology" OR "prenatal nutritional physiological phenomena" OR "prenatal nutritional physiological phenomenon" )

Concept:

( TITLE-ABS-KEY ( child* OR adolescen* OR neonat* OR kid* OR infant OR youth OR ( child* AND development ) OR ( adolescen* AND development ) OR ( neonat* AND development ) OR ( infant AND development ) OR (y outh AND development ) ) ) AND ( TITLE-ABS-KEY ( neurodevelopment* OR "nerve cell differentiation" OR cogniti* OR "cognitive development" OR function* OR autis* OR "autism spectrum disorder*" OR "prevasive developmental disorder*" OR "attention deficit hyperactivity disorder" OR "adhd" OR "mental performance" OR ( language AND development ) OR "language disability" OR "language disorder" OR "developmental language disorder" OR ( motor AND development ) OR ( motor AND skills ) OR "psychomotor development" OR "psychomotor evolution" OR ( psychosocial AND function* ) OR ( emotio* AND development ) OR "executive function" OR "intelligence quotient" OR iq OR "iq score" OR i.q. OR i.q OR "intellectual quotient" OR "working memory index" OR "processing speed index" OR "perceptual reasoning index" OR "verbal comprehension index" OR "wechsler intelligence scale" OR "bayley scales of infant development" OR "development* neurology" OR ( development* AND milestone* ) OR "disorder* of higher cerebral function" OR ( cogniti* AND development ) ) )

Context:

(aruba) OR (caribbean netherlands) OR (curacao) OR (saint martin) OR (antigua and barbuda) OR (bahamas) OR (barbados) OR (virgin islands) OR (cuba) OR (cuban) OR (dominica) OR (dominican republic) OR (dominican*) OR (grenada) OR (guadeloupe) OR (haiti) OR (haitian*) OR (jamaica) OR (jamaican) OR (martinique) OR (puerto rico) OR (puerto rican*) OR (saint kitts and nevis) OR (saint lucia) OR (saint vincent and the grenadines) OR (trinidad and tobago) OR (virgin island*) OR (caribbean) OR (caribbean region) OR (caribbean islands) OR (west indies)
